# Prognostic value of ictal onset patterns in postsurgical outcome of temporal lobe epilepsy

**Published:** 2017-10-07

**Authors:** Jafar Mehvari-Habibabadi, Reza Basiratnia, Houshang Moein, Mohamad Zare, Majid Barakatain, Yahya Aghakhani, Nasim Tabrizi

**Affiliations:** 1 Isfahan Neurosciences Research Center, Isfahan University of Medical Sciences, Isfahan, Iran; 2 Department of Clinical Neurosciences, University of Calgary, Calgary, Canada; 3 Isfahan University of Medical Sciences, Isfahan, Iran; 4 Department of Neurology, School of Medicine, Mazandaran University of Medical Sciences, Sari, Iran

**Keywords:** Electroencephalography, Temporal Lobe, Epilepsy, Surgery, Outcome

## Abstract

**Background:** To investigate ictal onset patterns (IOP) in scalp electroencephalography (EEG) of patients with temporal lobe epilepsy (TLE) and their prognostic effect on the postoperative outcome.

**Methods:** We conducted a retrospective cohort study between 2011 and 2015 in our referral Epilepsy Surgery Center enrolling adult patients with refractory TLE and a visible epileptogenic lesion in magnetic resonance imaging (MRI), who underwent epilepsy surgery. Demographic, clinical and MRI findings were collected and ictal findings during video-EEG monitoring were reviewed in detail. The correlation between preoperative findings and the postsurgical outcome was analyzed.

**Results:** We reviewed 303 seizures in 93 patients. Rhythmic theta and rhythmic spike/sharp and wave were respectively the most common initial ictal pattern and late significant discharges. Engel class I outcome was observed in 88.2% of patients. Female sex, aura, the absence of secondary generalization, rhythmic theta as initial ictal pattern and concordance of ictal-interictal EEG findings were correlated with favorable 1-year postsurgical outcome.

**Conclusion:** Preoperative clinical and EEG findings can provide valuable information regarding postsurgical prognosis in TLE patients.

## Introduction

The surgical treatment is currently a reasonable approach for patients with focal drug-resistant epilepsy. With the aim of precise determination of epileptogenic zone, presurgical evaluation often consists of careful analysis of seizure semiology, interictal and ictal electroencephalography (EEG), magnetic resonance imaging (MRI), neuropsychological tests and sometimes functional imaging, positron emission tomography (PET) and single photon emission computed tomography (SPECT).^1^ However, despite all the investigations, surgical processes have shown variable success rates and many prognostic factors have been proposed.^2^^-^^7^

Prolonged video-EEG monitoring is still the cornerstone of presurgical evaluation. Most studies on EEG, as a prognostic factor of epilepsy surgery, have investigated the ability of interictal and ictal EEG to lateralize and localize the irritative and seizure onset zone and its concordance with clinical and imaging findings.^8^^-^^11^ There are also studies on correlation of postoperative EEG discharges with postoperative outcome.^12^^-^^14^ However, few surveys investigated the prognostic value of ictal EEG patterns in epilepsy surgery. Some of them have confirmed the correlation between ictal onset patterns (IOP) and surgery outcome^15^^-^^17^ and some have failed to find any prognostic value for IOPs.^2^^,^^18^^-^^20^ Sample size in most of these studies is small and the results are controversial. Another problem that makes it difficult to draw a conclusion is the evaluation of one seizure per patient in some studies, which possibly accompanies by the unwanted consequence of selection bias. Therefore, it remains unclear if the detailed analysis of IOP under certain circumstances can help in determining the postsurgical outcome. In this study, we evaluated all recorded seizures for each patient in order to investigate the prognostic value of IOPs in the postsurgical outcome of patients with temporal lobe epilepsy (TLE).

## Materials and Methods

This retrospective cohort study was conducted at the referral Epilepsy Surgery Center of Isfahan Medical School University Hospital, Iran, between September 2011 and December 2015. We searched the database and enrolled the adult patients with medically refractory TLE who were admitted for presurgical evaluation. All patients underwent noninvasive long-term video-EEG monitoring until the epileptogenic zone could be definitely determined and it took about 3 to 11 days for different patients. They consequently underwent epilepsy surgery. The inclusion criteria were defined as the age of 18-60 years, MRI evidence of lesion relevant to epileptogenic zone, and one-year follow-up. We excluded patients with prior epilepsy surgery, inadequate data and follow-up evidence of less than 1 year. 93 patients met the criteria. This study was approved by the Ethics Board of Isfahan University of Medical Sciences.

Data including age, sex, handedness, marital status, age at seizure onset, seizure semiology and frequency, risk factors for epilepsy, family history of epilepsy (according to patients’ history), irritative zone (based on interictal EEG data), pre-operative MRI findings, type and side of surgery and pathological findings were gathered based on patients’ records.

All patients had prolonged scalp EEG monitoring using Nihon Kohden system. Electrodes arranged according to the International 10–20 system with additional temporal electrodes (F9, F10, T9, T10, T1 and T2). The setting was arranged at 200 Hz sampling rate, 0.1 second time constant and 60 Hz notch filter.

We retrospectively reviewed recorded data from 93 patients with 303 seizures. The ictal EEGs that were significantly contaminated by the artifact and rare seizures which occurred when the patient was disconnected from recording set were excluded. We analyzed all registered seizures for each patient. If the patient had more than 10 seizures, we considered the first 10 appropriate ones. At first, ictal rhythms were studied in longitudinal bipolar and referential montages with common filtration [low-frequency filter (LFF) 1 Hz, high-frequency filter (HFF) 70 Hz]. During the evaluation, digital filtering and gain were adjusted to improve the EEG display. 

We assessed ictal scalp EEG in four steps. First, we registered the time of onset, awake/asleep state of patient and semiology of each seizure.^21^ Then, we noted the interval between clinical manifestations and ictal EEG onset. If there was an aura before other clinical signs, time of clinical presentation was accepted at patient’s notification. Duration of artifacts and their time of onset during ictal patterns were also registered. In next step, we described the morphology of ictal discharges during seizure including fast activity (FA), rhythmic alpha activity (RA), rhythmic theta activity, rhythmic delta activity (RD), rhythmic spike/sharp and wave (RS/Sh and W) and background attenuation (BA). We described ictal patterns as a pattern at onset (PAO) and late significant pattern (LSP). The PAO was defined as first definite ictal EEG changes which associated or followed by a clinical seizure. Any changes in morphology or location of PAO was considered as LSP and numbered consecutively. Finally, we noted duration of seizure, occurrence and time of ipsilateral and contralateral propagation of epileptic discharges. We defined ictal-interictal match as ≥ 90% predominance of interictal discharges at the side of the epileptogenic zone.

The ictal EEG findings of all patients were investigated by an epilepsy fellowship assistant and were reviewed by an expert epileptologist. The electroencephalographers were blinded for interictal EEG findings and MRI results.

We collected 1-year follow-up results from registered post-operative outpatient visits and classified them as favorable and unfavorable. The favorable outcome encompassed class I of Engel outcome scale^22^ and the unfavorable outcome was defined as class II-IV.

Statistical analysis was performed using SPSS software (version 23, IBM Corporation, Armonk, NY, USA). Continuous variables were presented as the mean ± standard deviation (SD), median (min-max) as appropriate. Qualitative variables were reported as number (percent). Before the main analysis, Shapiro-Wilk test was performed to check normality. Student's independent t-test, chi-square test, Fisher’s exact test, Mann-Whitney test and analysis of variance (ANOVA) were used. We applied Cohen's Kappa statistic to assess inter-observer agreement. All probability tests were two-tailed and the level of significance was defined as P ≤ 0.050.

## Results

We retrospectively reviewed 303 seizures in 93 patients. 303 seizures were analyzed according to defined method. Mean reviewed seizure numbers for each patient was 3.2 ± 1.8. Patients’ characteristics are summarized in table 1.

***Clinical seizure data:*** From the semiologic point of view, 79.9% of seizures were categorized in motor type, of which 72.2% were classified as a complex motor seizure (70.9% automotor, 1.3% hypermotor) and 7.7% simple motor seizure. 20.1% of seizures were dialeptic. Aura was detected prior to 14.9% of clinical seizures and 14.2% of seizures were progressed to secondary generalization. 35.1% of seizures occurred during sleep.

***Ictal EEG findings:*** EEG changes were started before and after clinical onset in 88 (29.0%) and 82 (27.1%) seizures, respectively. Simultaneous occurrence of IOPs and clinical signs were detected in 133 (43.9%) seizures. In 10 seizures the exact correlation could not be determined due to artifact at onset. The median interval of EEG changes before and after clinical signs was 4 (1-40) and 6.5 (1-29) seconds. Presence of EEG changes before and simultaneous to clinical signs had a significant correlation with localization ability of PAO (P = 0.020), but not the next patterns.

Mean total duration of seizures was 74.7 ± 48.4 seconds (range: 7-464). Propagation to ipsilateral hemisphere occurred in 97.0% of localizing IOPs with a mean time of 6.1 seconds (0.5-38). Lateralization switch was detected in 1.6% of seizures with a mean time of 52.8 seconds (Min-max: 5-100) from seizure onset. Contralateral propagation appeared in 94.9% of primarily lateralized seizures and the mean time of contralateral propagation was 10.2 seconds (0.5-342), although the accurate time of contralateral propagation remained unclear in 33 seizures because of overlapping artifacts.

**Table 1:** Patients’ characteristics

**Characteristic**		**Value**
Sex (%)	Man	52.7
	Woman	47.3
Handedness (%)	Right	89.2
	Left	10.8
Marital status (%)	Single	62.4
	Married	37.6
Age at surgery (year) (mean ± SD)		28.9 ± 8.9
Age of onset (year) (mean ± SD)		10.5 ± 8.4
Seizure Frequency [n (%)]	Daily	15 (16.1)
	Weekly	58 (62.4)
	Monthly	13 (14)
	Seasonal	7 (7.5)
Epilepsy duration (year) (mean ± SD)		18.5 ± 11.1
Number of AEDs (mean ± SD) (range)		2.6 ± 1 (0-5)
Risk factors [n (%)]	Perinatal complication	9 (9.7)
	Febrile convulsion	24 (25.8)
	CNS infection	4 (4.3)
	Developmental delay	6 (6.5)
	Head trauma	26 (28.0)
Family history (%)	Positive	8.6
	Negative	91.4
Side of epileptogenic zone (%)	Right	47.3
	Left	52.7
Pathological Findings [n (%)]	HS	53 (57)
	Tumor	18 (19.4)
	Gliosis	16 (17.2)
	FCD	4 (4.3)
	CA	2 (2.2)
Engel’s surgical outcome [n (%)]	Engel I (favorable)	82 (88.2)
	Engel II, III, IV (unfavorable)	11 (11.8)

SD: Standard deviation; AED: Antiepileptic drug; CNS: Central nervous system; HS: Hippocampal sclerosis; FCD: Focal cortical dysplasia; CA: Cavernous angioma

***PAO:*** In patients with more than one reviewed seizures, similar PAO morphology was observed in 41.5%, while 2 to 3 different PAO morphologies were detected in 58.5%. The most common PAO morphology was rhythmic theta. Concerning semiology, rhythmic theta was the most common PAO morphology in automotor seizures (54.2%) (P = 0.040) and occurred frequently in dialeptic seizures and simple motor seizures as well. However, the most frequent PAO in hypermotor seizures was RD. PAO morphology had no significant correlation with localization ability, but rhythmic theta was significantly relevant to seizure lateralization (P = 0.010).

Presentation of rhythmic theta as PAO was significantly more common in awake state (P = 0.010) and RS/Sh and W was the most frequent PAO at sleep (41.2%). PAO characteristics had no correlation with clinically secondary generalization, the time interval between EEG changes and clinical signs and ipsilateral or contralateral propagation of ictal discharges.

***LSP1:*** LSP1 was detected in 224 (73.9%) seizures. RS/Sh and W was the most common LSP1 morphology in spite of semiology and was associated with the highest lateralization ability (P = 0.030). LSP1 could detect the correct side and location of seizure onset in 60.9% and 24.4% of seizures with unsuccessful lateralization and localization by PAO. No significant correlations were detected between LSP morphology and ipsilateral or contralateral propagation, localization and sleep/waking state.

***LSP2:*** LSP2 occurred in 56 (18.5%) seizures. Lateralization and localization by LSP2 had no significant difference between different semiologies, sleep/wake state and time of seizure onset. More characteristics are summarized in table 2.

***Surgery and pathology findings:*** The surgery types were selective amygdalohippocampectomy (SAH) in 60.9% and lesionectomy in 39.1% of patients. Pathological findings are summarized in table 1.

***Surgery outcome:*** Based on Engel criteria, 1-year follow-up surgery outcome was favorable (class I) in 88.2% of patients and the unfavorable outcome was detected in 11.8% of patients (8.8% class II, 2.2% class III and 2.2% class IV). 

***Patient characteristics and outcome:*** Despite the similar distribution of prognostic factors in men and women TLE patients, the outcome was significantly better in females with TLE (P = 0.007). Handedness, marital status, number of AEDs, age at epilepsy onset, age at surgery, duration of epilepsy, seizure frequency, family history and risk factors of epilepsy had no prognostic value for the outcome (Table 3).

***Seizure characteristics and outcome:*** Presence of aura had a significant association with favorable outcome (P = 0.050). The outcome was significantly better in automotor seizures (P = 0.010), while hypermotor seizures had the worst postsurgical outcome (50.0%). Patients who experienced secondarily generalized seizures had a less favorable outcome (P = 0.030). The occurrence of sleep-related seizures had no effect on outcome (P = 0.520).

***EEG characteristics and outcome:*** Rhythmic theta as PAO was associated with significantly better outcome (P = 0.030). To obtain a better knowledge of the effect of rhythmic theta as PAO on the outcome, we more precisely analyzed the characteristics of patients with this PAO and unfavorable outcome. The pathological findings in those patients who had rhythmic theta as PAO, but experienced unfavorable outcome, were hippocampal sclerosis (HS) (87.5% vs. 66.7% in favorable outcome) and focal cortical dysplasia (FCD) (12.5% vs. 1.3% in favorable). The most common seizure semiology in this group was dialeptic (50.0% vs. 17.2% in favorable outcome). There was no considerable difference regarding ipsilateral and contralateral propagation, lateralization and localization by PAO, ictal EEG/clinical seizure interval or clinically secondary generalization.

**Table 2:** Ictal onset patterns (IOP)

**Characteristic**		**PAO**	**LSP1**	**LSP2**
Morphology [n (%)]	Rhythmic theta	161 (53.1)	39 (17.4)	6 (10.7)
	RD	45 (14.9)	24 (10.7)	10 (17.9)
	RS/Sh and W	34 (11.2)	145 (64.7)	35 (62.5)
	Other rhythms (RA, FA, BA)	63 (20.8)	16 (7.1)	5 (8.9)
Duration (s) [median (min-max)]		13 (0.5-120)	32 (1-273)	34.5 (3-196)
Lateralization [n (%)]		228 (75.5)	132 (60)	29 (51.8)
Mean lateralization per patient		78.9	58.5	54.8
Localization [n (%)]		129 (56.1)	53 (40.2)	8 (27.6)
Mean localization per patient		49.9	21.1	15.6

PAO: Pattern at onset; LSP: Late significant patterns; RD: Rhythmic delta; RS/Sh and W: Rhythmic spike/sharp and wave; RA: Rhythmic alpha; FA: Fast activity; BA: Background attenuation

**Table 3:** Predictors of postsurgical outcome

**Variable**		**Outcome**		**P** ^*^
		**Engel I**	**Engel II, III, IV**	
Age at onset (year)	≤ 5	28	1	0.080
	> 5	53	10	
Age at surgery (year)	≤ 35	66	8	0.540
	> 35	16	3	
Sex	Man	39	10	0.007
	Woman	43	1	
Epilepsy duration (year)	> 15	44	6	0.950
	≤ 15	38	5	
AED	≤ 3	70	8	0.280
	> 3	12	3	
Frequency	Daily	14	1	0.510
	Weekly	49	9	
	Monthly	12	1	
	Seasonal	7	0	
Seizure semiology (automotor)		196	16	0.010
Aura		44	1	0.050
Secondary generalization		35	8	0.030
EEG vs. clinical onset	Before	77	11	0.460
	Simultaneous	123	10	
	After	74	8	
PAO morphology (rhythmic theta)		153	8	0.030
Lateralization by PAO		205	23	0.610
LSP1 morphology (RD)		23	1	0.400
Lateralization by LSP1		51	2	0.080
Contralateral propagation		199	25	0.760
Ictal-interictal match		64	6	0.010

AED: Antiepileptic drug; EEG: Electroencephalography; PAO: Pattern at onset; LSP: Late significant pattern; RD: Rhythmic delta

Back to the original data for all patients, we realized that LSP1 and LSP2 morphology had no significant correlation with surgery outcome (P = 0.400 and P = 0.780 respectively). Patients with an ictal-interictal match (75.3%) were found to have the significantly better outcome (P = 0.010).

The outcome had no correlation with the intra-individual lateralizing or localizing seizure proportion (P = 0.490 and P = 0.550). We also failed to find any correlation between the ictal EEG/clinical seizure interval, the presence of multiple PAOs per patient, ipsilateral and contralateral propagation of IOPs or their time of onset and outcome (Table 3).

**Surgery, pathology and outcome**

Pathological findings, surgery side, and type had no significant effect on the outcome.

**Inter-observer reliability**

Inter-observer reliability for lateralization of IOPs was excellent (κ = 0.92 for PAO and κ = 0.96 for LSP, P < 0.001) and inter-observer agreement for localization was also perfect (κ = 0.89 for PAO and κ = 0.91 for LSP, P < 0.001).

## Discussion

This retrospective cohort study was designed to evaluate ictal patterns in patients with refractory TLE who underwent surgery as well as investigating their prognostic value in surgical outcome. Unlike most similar studies, we investigated all recorded seizures for each patient during LTM to prevent neglecting different IOPs and semiologies that could occur in each patient. This approach seems necessary because two or more distinct PAOs have been detected in more than half of our patients and also about one-fifth of them have experienced different seizure semiologies.

Reviewing all enrolled patients, in line with most of the similar studies, we failed to find any correlation between age at onset of epilepsy,^2^^,^^20^^,^^23^^,^^24^ age at surgery,^2^^,^^20^^,^^23^^,^^24^ duration of epilepsy,^2^^,^^20^^,^^23^^-^^26^ preoperative seizure frequency^2^^,^^27^^,^^28^ and risk factors of epilepsy^23^^,^^28^^-^^30^ with the outcome. Despite the few studies reporting man sex as a negative prognostic factor in epilepsy surgery,^31^ most of the studies did not find sex as a factor influencing the postsurgical outcome.^2^^,^^18^^,^^20^^,^^24^^,^^32^ In contrast to similar studies, we have found the better postsurgical outcome in our female patients. Janszky, et al.^33^ have mentioned more secondary generalization in man and more isolated auras and lateralized seizure patterns in woman patients with mesial TLE, which can somehow explain better postoperative outcome in female patients. Nevertheless, we did not find any significant difference between man and woman patients regarding above and other prognostic factors.

In our study, the presence of aura was associated with more favorable outcome. This finding can reflect the fact that most of the reported auras in our study occurred in mesial TLE patients with expected better outcome. Although we could not find any correlation between the contralateral propagation of epileptic discharges and outcome, similar to previous findings,^23^^,^^27^^-^^30^^,^^34^ our patients with contralateral propagation which experienced secondarily generalized seizures had significantly unfavorable outcomes. We suggest that contralateral propagation of epileptic discharges, in a way that causes a clinically generalized seizure, is needed to affect the outcome. The underlying mechanism is probably a more diffuse epileptogenic zone or predisposition to secondary epileptogenesis.

According to the results of previous studies,^15^^,^^20^^,^^35^ rhythmic theta was the most frequent initial ictal discharge in our patients. Our review of ictal findings showed that regardless of other factors, rhythmic theta as PAO is significantly associated with favorable outcome in TLE. In line with our results, Sirin, et al.^16^ have shown a correlation between rhythmic theta/alpha activity and postsurgical seizure freedom in TLE, although they did not distinct theta and alpha rhythms which make the comparison imprecise. Furthermore, Assaf and Ebersole,^15^ in their survey on ictal EEG predictors of postsurgical outcome in TLE, have shown that theta rhythm as ictal onset pattern on visual scalp EEG predicts a significantly better outcome than other rhythms. Also, Lau, et al.^35^ in their study on TLE surgery remarked theta rhythm as the most common ictal pattern that carries the best prognosis for TLE particularly in those who have evidence of HS in MRI. In contrast, Malter, et al.^20^ in their recent study on the predictive value of scalp EEG for epilepsy surgery did not find any correlation between initial ictal patterns and postoperative outcome in TLE. In a similar study by Monnerat, et al.^2^ authors indicated that ictal EEG patterns could not provide any prognostic information regarding the postsurgical outcome, though they did not provide any details about the morphology of IOPs. Although lateralization by PAO and LSP1 was commonly possible in our patients, we found that correct lateralization is more important than the proportion of lateralizing seizures in each patient. The most lateralizing initial pattern in our patients was rhythmic theta and when it presented as PAO, it was considerably correlated to automotor semiology. Our results confirmed previous reports of better postoperative outcome in the case of an ictal-interictal match.^8^^,^^9^ Since we merely enrolled patients with MRI-visible lesions in this study, our results could only be extended to this group of patients.

## Conclusion

Our findings have shown that in TLE patients, woman sex, the presence of aura compatible with mesial temporal lobe origin, the absence of secondary generalization, rhythmic theta as PAO and concordance of ictal-interictal EEG findings are associated with a more favorable outcome.

## Notes:

**How to cite this article:** Mehvari-Habibabadi J, Basiratnia R, Moein H, Zare M, Barakatain M, Aghakhani Y, et al. Prognostic value of ictal onset patterns in postsurgical outcome of temporal lobe epilepsy. Iran J Neurol 2017; 16(4): 185-91.
